# Isomerization of *trans*‐3‐methylglutaconic acid

**DOI:** 10.1002/jmd2.12185

**Published:** 2020-11-11

**Authors:** Dylan E. Jones, J. David Ricker, Laina M. Geary, Dylan K. Kosma, Robert O. Ryan

**Affiliations:** ^1^ Department of Biochemistry and Molecular Biology University of Nevada Reno Reno Nevada USA; ^2^ Department of Chemistry University of Nevada Reno Reno Nevada USA

**Keywords:** 3‐methylglutaconic aciduria, *cis*:*trans* isomerization, gas chromatography‐mass spectrometry, inborn errors of metabolism, mitochondria, NMR spectroscopy

## Abstract

3‐Methylglutaconic (3MGC) aciduria is a common phenotypic feature of a growing number of inborn errors of metabolism. “Primary” 3MGC aciduria is caused by deficiencies in leucine pathway enzymes while “secondary” 3MGC aciduria results from inborn errors of metabolism that impact mitochondrial energy production. The metabolic precursor of 3MGC acid is *trans*‐3MGC CoA, an intermediate in the leucine catabolism pathway. Gas chromatography‐mass spectrometry (GC‐MS) analysis of commercially available *trans*‐3MGC acid yielded a mixture of *cis* and *trans* isomers while ^1^H‐NMR spectroscopy of *trans*‐3MGC acid at 25°C provided no evidence for the *cis* isomer. When *trans*‐3MGC acid was incubated under conditions used for sample derivatization prior to GC‐MS (but with no trimethylsilane added), ^1^H‐NMR spectroscopy provided evidence of *trans* to *cis* isomerization. Incubation of *trans*‐3MGC acid at 37°C resulted in time‐dependent isomerization to *cis*‐3MGC acid. *Cis*‐3MGC acid behaved in a similar manner except that, under identical incubation conditions, less isomerization occurred. In agreement with these experimental results, molecular modeling studies provided evidence that the energy minimized structure of *cis*‐3MGC acid is 4 kJ/mol more stable than that for *trans*‐3MGC acid. Once generated in vivo, *trans*‐3MGC acid is proposed to isomerize via a mechanism involving π electron delocalization with formation of a resonance structure that permits bond rotation. The data presented are consistent with the occurrence of both diastereomers in urine samples of subjects with 3MGC aciduria.

**Abbreviations**
3MGC: 3‐methylglutaconic
AUH: 3‐methylglutaconyl CoA hydratase
BSTFA‐TCMS: bis(trimethylsilyl)trifluoroacetamide‐trichloromethylsilane
D_2_O: deuterium oxide
DMSO: dimethylsulfoxide
ETC: electron transport chain
GC‐MS: gas chromatography‐mass spectrometry
HMG: 3‐hydroxy‐3‐methylglutaryl
HMGCL: 3‐hydroxy‐3‐methylglutaryl CoA lyase
TCA: tricarboxylic acid cycle

**Synopsis**

Isomerization of *trans*‐3‐methylglutaconic acid occurs spontaneously as a function of time and temperature, explaining the presence of both isomers in 3‐methylglutaconic acidurias.

## 1 INTRODUCTION

3‐Methylglutaconic (3MGC) acid accumulates in urine of individuals suffering from a wide variety of inborn errors of metabolism. Two categories of 3MGC aciduria have been identified.^1^ In primary 3MGC aciduria, mutations in genes encoding either of two leucine metabolic pathway enzymes results in a buildup of upstream intermediates and excretion of 3MGC acid. Specifically, deficiencies in 3MGC CoA hydratase (*AUH*) or 3‐hydroxy‐3‐methylglutaryl (HMG) CoA lyase (*HMGCL*) are associated with 3MGC aciduria.^2^ In leucine metabolism, AUH catalyzes hydration of *trans*‐3MGC CoA to (*S*)‐HMG CoA while HMG CoA lyase cleaves (*S*)‐HMG CoA to acetoacetate plus acetyl CoA. When either of these enzymes is deficient, leucine administration results in increased urinary excretion of 3MGC acid.^3^ Normally, during leucine catabolism the pathway intermediate 3‐methylcrotonyl CoA is carboxylated to form *trans*‐3MGC CoA which is hydrated to HMG CoA by AUH. When a deficiency occurs in AUH or HMGCL, *trans*‐3MGC CoA accumulates and, above a threshold, undergoes thioester hydrolysis, generating free CoASH and *trans*‐3MGC acid, which is excreted in urine.

Unlike primary 3MGC aciduria, no leucine pathway enzyme deficiencies exist in secondary 3MGC aciduria. Moreover, leucine loading has little effect on the amount of 3MGC acid excreted in urine.^3^ These features indicate that secondary 3MGC aciduria arises from an alternate biosynthetic route. In every case, secondary 3MGC aciduria is associated with mutations in genes encoding proteins that, directly or indirectly, affect mitochondrial energy metabolism. By interfering with the efficiency of aerobic respiration, electron transport chain (ETC) activity, and oxidative phosphorylation, are compromised. Under these circumstances, in cardiac and skeletal muscle mitochondria, NADH levels rise, the TCA cycle is inhibited and, in three steps, acetyl CoA is diverted to 3MGC CoA.^2^ The enzymes involved include methylacetoacetyl‐CoA thiolase, HMG CoA synthase 2 and AUH, which functions in the reverse direction, dehydrating (*S*)‐HMG CoA to *trans*‐3MGC CoA. When formed under these conditions, *trans*‐3MGC CoA has few options and, ultimately, some portion undergoes thioester hydrolysis to *trans*‐3MGC acid, which is excreted in urine.

By either biosynthetic route, 3MGC CoA is formed exclusively as the *trans* diastereomer. Based on this it may be anticipated that, upon analysis of urine organic acids, *trans*‐3MGC acid should be the sole diastereomer present. However, in nuclear magnetic resonance (NMR) studies, Iles et al^4^ and Engelke et al ^5^ reported an approximate 2:1 ratio of *cis*/*trans‐*3MGC acid in urine of individuals with primary 3MGC aciduria. In a similar manner, organic acid analysis by gas chromatography‐mass spectrometry (GC‐MS) of urine samples from subjects with secondary 3MGC aciduria revealed a mixture of *cis‐* and *trans‐*3MGC acid.^6^ Moreover, when diastereomerically pure *trans*‐3MGC acid was analyzed by GC‐MS, a 60:40 *cis*:*trans* ratio was observed.^7^

Given that no chemical or biological explanation exists for the apparent isomerization of *trans*‐3MGC acid, studies were conducted to investigate this phenomenon. Using commercially available *trans‐* and *cis*‐3MGC acid standards, GC‐MS and one dimensional ^1^H‐NMR spectroscopy experiments were conducted. The results obtained reveal that isomerization is dependent on temperature, time and chemical environment and that isomerization of *trans*‐3MGC acid to *cis*‐3MGC acid occurs more readily than isomerization of *cis*‐3MGC acid to *trans*‐3MGC acid at both physiological and elevated temperatures. These results, together with molecular modeling studies, support the conclusion that *cis*‐3MGC acid is the more stable diastereomer. The activation energy barrier for isomerization is likely overcome under relatively mild conditions by alkene π electron delocalization that permits C2‐C3 bond rotation.

## 2 MATERIALS AND METHODS

### 2.1 Chemicals and reagents

Synthetic standards of *cis*‐ and *trans*‐3MGC acid (HPLC grade, >97% pure) were purchased from Millipore‐Sigma and used without further modification. Deuterated solvents (D_2_O and DMSO‐*d*_6_) used for ^1^H‐NMR experiments were from Millipore‐Sigma. *N*,*O*‐Bis(trimethylsilyl)trifluoroacetamide‐trichloromethylsilane (BSTFA‐TCMS, 99:1) was from TCI Chemicals and anhydrous pyridine (>99%) was from Chem‐Impex.

### 2.2 Incubation, extraction, and sample preparation of 3MGC acid

Samples containing *cis*‐ or *trans‐*3MGC acid were extracted from acidified aqueous media with ethyl acetate and dried under a stream of N_2_ gas. For ^1^H‐NMR spectroscopy, the dried extract was dissolved in 600 μL D_2_O or DMSO‐*d*_6_. For GC‐MS analysis, extracts were dissolved in 100 μL anhydrous pyridine plus 100 μL BSTFA:TCMS (99:1) and incubated for 30 minutes at 70°C. The samples were cooled, dried under a stream of N_2_ gas and dissolved in acetonitrile.

### 2.3 GC‐MS analysis

Silylated 3MGC acid was applied to an Agilent 7890 GC equipped with a 5977 mass spectrometer detector (MSD) and flame ionization detector (FID). A 30‐m DB‐5 ms GC column connected with a 10‐m DuraGuard column (250 μm i.d. 0.250 μm film thickness) was used as the primary column. Samples (2 μL in acetonitrile) were injected via splitless injection. Injector temperature was held at 230°C. Column flow rate (He) was a constant 2 mL/min. The GC oven was programmed with an initial temperature of 80°C that was increased by 10°C/min to 270°C and held for 10 minutes. Column eluent was directed to an FID electronic pressure control (EPC) flow control manifold that splits the flow to the FID and MSD (~1:1 ratio). Columns running from the EPC to the FID (1.7 m, 18 μm i.d.) or MSD (1 m, 200 μm i.d.) were comprised of deactivated fused silica. FID temperature was set to 240°C. MSD transfer line was set to 280°C. MSD parameters: EI 70 eV, m/z 40‐700, 2 scans s^−1^. Data were analyzed using Agilent MassHunter Qualitative Analysis software.

### 2.4 NMR spectroscopy

^1^H‐NMR (400 MHz) spectra were obtained on a Varian 400 spectrometer. All chemical shifts for *cis‐* and *trans*‐3MGC acid are in ppm units relative to solvent resonances: H_2_O: 4.8 ppm, DMSO‐*d*_5_ = 2.50 ppm. ^1^H‐NMR spectra were processed using MestReNova software. The ratio between *cis*:*trans* diastereomers was calculated by integration of the methyl proton resonances of each isomer. Variable‐temperature ^1^H‐NMR experiments were conducted by adjusting the probe temperature between 5°C and 95°C. When the desired probe temperature was reached, 24 scans were collected, the probe temperature was increased by 10°C and another 24 scans collected. For constant temperature ^1^H‐NMR experiments, the probe was heated to a specified temperature and maintained for indicated times prior to acquisition of spectra.

### 2.5 Molecular modeling of *cis*‐ and *trans*‐3MGC acid

Electronic structure calculations were performed using the electronic structure package GAMESS.^8^ All calculations utilized Ahlrich's def2‐tzvp basis set^9^ obtained from the basis set exchange,^10^, ^11^, ^12^ and B3LYP^13^, ^14^, ^15^, ^16^ hybrid density functional. Numerous input geometries yielded optimized structures. These optimized geometries were verified as stationary points on the potential energy surface by Hessian calculations.

## 3 RESULTS

### 3.1 GC‐MS analysis of *trans*‐ and *cis*‐3MGC acid standards

**FIGURE 1:**
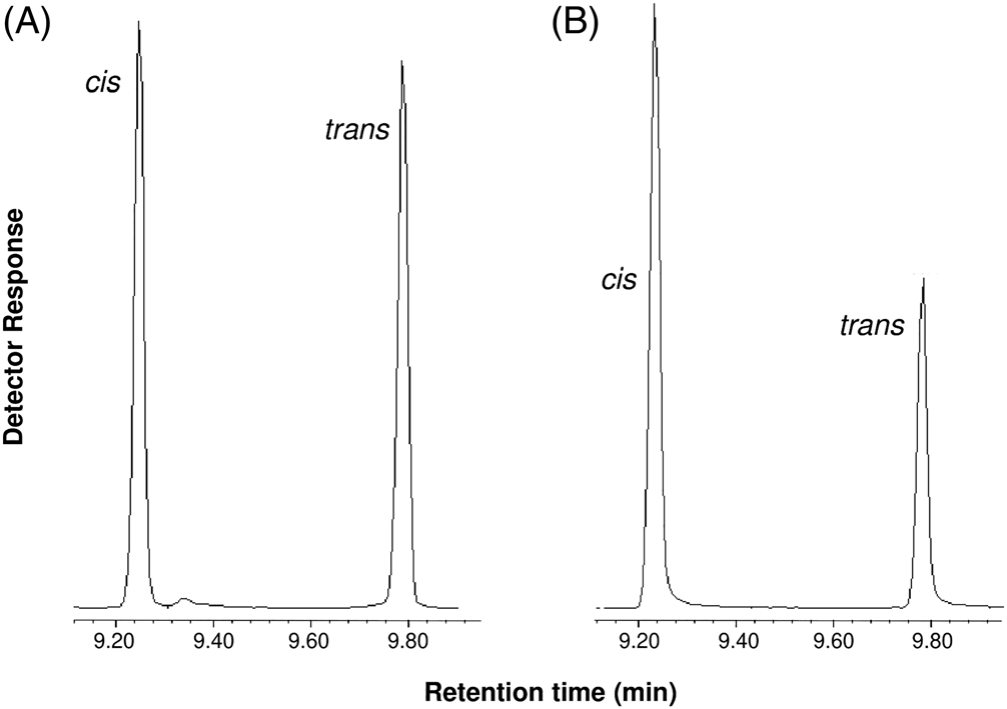
GC–MS analysis of 3MGC acid standards. *Trans*‐3MGC acid and *cis*‐3MGC acid standards (>97% isomeric purity for each) were derivatized with trimethylsilane (TMS) as described in Materials and Methods. The resulting TMS derivatives were dissolved in acetonitrile and 2 μL injected onto a Agilent 7890 GC equipped with a 5977 mass spectrometer detector and flame ionization detector. A, GC trace of the TMS derivative of *trans*‐3MGC acid and B, GC trace of the TMS derivatize of *cis*‐3MGC acid

Upon GC‐MS analysis of *trans‐* and *cis*‐3MGC acid standards, each diastereomer gave rise to two distinct peaks. Mass spectrometry analysis confirmed that these peaks correspond to *cis‐* and *trans*‐3MGC acid, respectively (Figure 1). This data confirms that *trans*‐3MGC acid is susceptible to isomerization,^6^, ^7^ and reveals that *cis*‐3MGC acid behaves in a similar manner. The isomer ratio in both samples favored the *cis* diastereomer. Insofar as sample preparation for GC‐MS involves trimethylsilylation of 3MGC acid, it is conceivable that the derivatization conditions employed, promote isomerization of diastereomerically pure *trans*‐ and *cis*‐3MGC acids. To investigate this further, ^1^H‐NMR spectroscopy, was employed.

### 3.2 ^1^H‐NMR spectroscopy of *trans*‐ and *cis*‐3MGC acid

**FIGURE 2:**
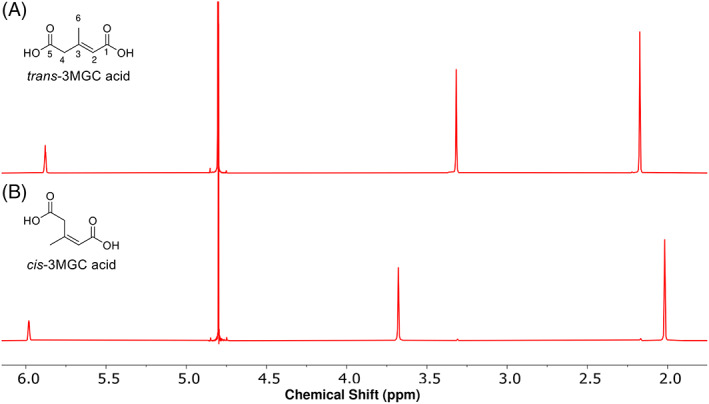
^1^H‐NMR spectroscopy of 3MGC acid. A *trans*‐3MGC acid standard, A, and a *cis*‐3MGC acid standard, B, were dissolved in D_2_O and ^1^H‐NMR spectra obtained at 25°C on a Varian 400 spectrometer. Chemical shifts are in ppm units relative to the D_2_O solvent resonance at 4.8 ppm, Spectra were processed using MestReNova software. Upper left corner of panels: chemical structures of *trans*‐ and *cis*‐3MGC acid with carbons numbered

^1^H‐NMR is a discriminating method that permits *cis* and *trans* diastereomers of 3MGC acid to be readily distinguished. When subjected to ^1^H‐NMR spectroscopy at 25°C in D_2_O, *trans*‐3MGC acid yielded a spectrum containing three distinct resonance peaks (chemical shifts = 5.86, 3.30, and 2.16 ppm). As reported previously,^4^, ^5^ these resonances correspond to the methyne proton (C2), methylene protons (C4), and the methyl protons of *trans*‐3MGC acid, respectively (Figure 2A). The resonance at ~4.8 ppm corresponds to residual H_2_O. A similar set of three resonances was observed in spectra of *cis*‐3MGC acid except that the chemical shifts were different (Figure 2B). The methyne proton shifted slightly downfield (5.86‐5.99 ppm) while the methylene protons showed a larger downfield shift (from 3.30 to 3.86 ppm). At the same time, the methyl protons shifted upfield (from 2.16 to 2.02 ppm). The observed chemical shifts and resonance assignments compare well with spectra reported earlier.^5^ Whereas *trans*‐3MGC acid gave rise to a diastereomerically pure spectrum, *cis*‐3MGC acid yielded a spectrum consistent with the presence of a small amount of *trans*‐3MGC acid (~3%; see minor resonances at 3.31 and 2.17 ppm).

### 3.3 Effect of sample preparation conditions on 3MGC acid isomerization

**FIGURE 3:**
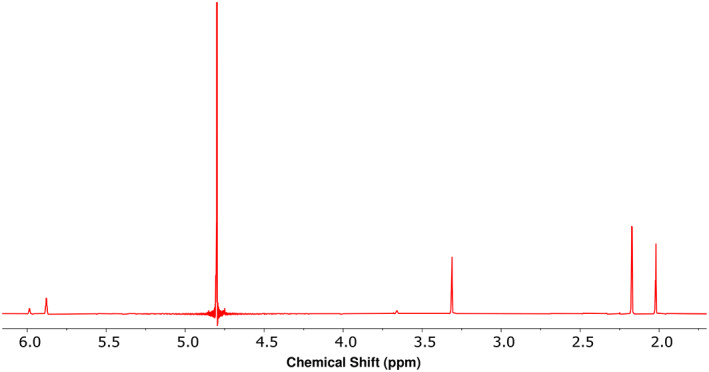
Effect of exposure to GC derivatization conditions on ^1^H‐NMR spectra of *trans*‐3MGC acid. A sample of *trans*‐3MGC acid was dissolved in H_2_O, acidified and extracted with ethyl acetate. The solvent was evaporated under a stream of N_2_ gas and the residue dissolved in pyridine and incubated at 70°C for 30 minutes. Following incubation, the solvent was evaporated and the residue dissolved in D_2_O and then analyzed by ^1^H‐NMR spectroscopy at 25°C. The relative abundance of each isomer was calculated by integration of the methyl proton resonance for *trans* (2.16 ppm) and *cis* (2.02 ppm) 3MGC acid, respectively

To determine the extent to which conditions used to convert *cis‐ and trans*‐3MGC acids to their corresponding TMS derivatives prior to GC‐MS analysis induces isomerization, ^1^H‐NMR experiments were performed. When *trans*‐3MGC acid was dissolved in pyridine and incubated at 70°C for 30 minutes in the absence of TMS, ^1^H‐NMR spectroscopy provided evidence of isomerization [Figure 3; see methyl proton resonances at 2.16 ppm (*trans*‐3MGC acid) and 2.02 ppm (*cis*‐3MGC acid)]. When *cis*‐3MGC acid was subjected to the same protocol isomerization was detected, although to a lesser extent than that observed with *trans*‐3MGC acid (data not shown). Thus, differences observed in the ratio of *cis* and *trans* diastereomers of 3MGC acid when analyzed by GC‐MS and ^1^H‐NMR spectroscopy can be attributed, at least in part, to the sample preparation conditions.

### 3.4 Effect of temperature and time on 3MGC acid isomerization

**FIGURE 4:**
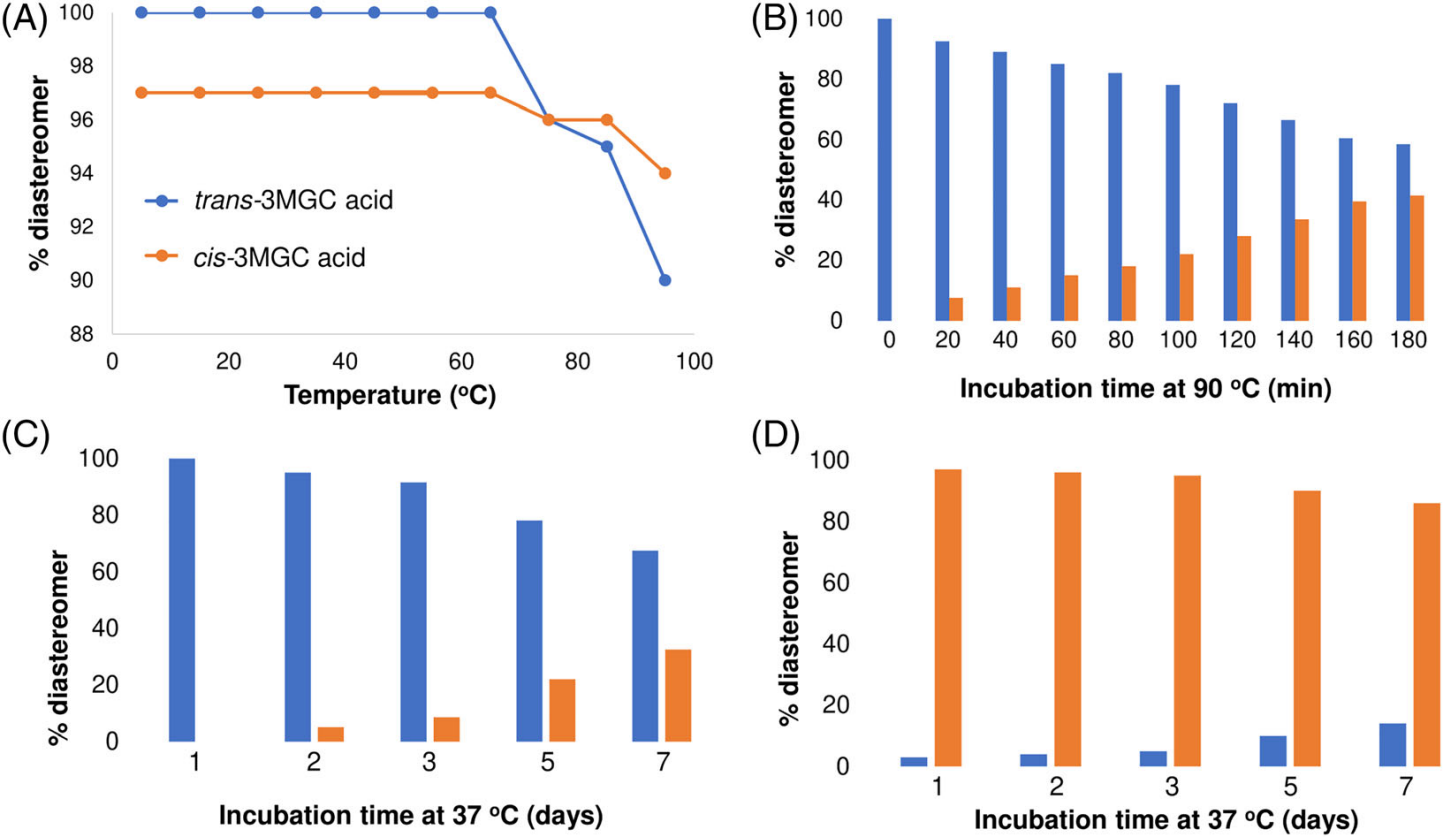
Effect of temperature and time on isomerization of 3MGC acid. A, Effect of temperature on isomerization of *trans*‐3MGC acid and *cis*‐3MGC acids. Samples of each organic acid (in D_2_O) were brought to a specified temperature in the NMR probe. As soon as a given temperature was reached, a ^1^H‐NMR spectrum was collected. The ratio of *trans* and *cis* isomers was determined from integration of the corresponding resonance peaks. B, Effect of incubation time at 90°C on isomerization of *trans*‐3MGC acid. A sample of *trans*‐3MGC acid, dissolved in D_2_O, was brought to 90°C and maintained at that temperature for specified times. At each time point a ^1^H‐NMR spectrum was collected and the ratio of *trans* and *cis* isomers present determined. C, Effect of incubation time at 37°C on isomerization of *trans*‐3MGC acid. A sample of *trans*‐3MGC acid in D_2_O was incubated for specified times at 37°C and, following incubation, ^1^H‐NMR spectra were collected and the ratio of *trans* and *cis* isomers present determined. D, Same as (c) except *cis*‐3MGC acid was used

To further investigate how temperature affects 3MGC acid isomerization, samples were analyzed by one dimensional ^1^H‐NMR. When spectra of *trans‐* or *cis*‐3MGC acid were collected immediately after the NMR probe reached a specified temperature, no isomerization occurred until ~70°C (Figure 4A). As the probe temperature was increased, however, isomerization was detected, with *trans*‐3MGC acid displaying a greater propensity to isomerize than *cis*‐3MGC acid. To investigate this further, the effect of incubation time at a constant temperature (90°C) on *trans*‐3MGC acid isomerization was examined. The data revealed that, after 180 minutes, the *trans:cis* ratio was ~60:40 (Figure 4B). Given that urine samples from subjects with primary 3MGC aciduria manifest a mixture of 3MGC acid diastereomers,^5^ the effect of incubation time at 37°C was investigated. Over the course of 7 days incubation, ^1^H‐NMR spectroscopy analysis provided evidence of *trans*‐3MGC acid isomerization (Figure 4C). When the same experiment was conducted using *cis*‐3MGC acid, less isomerization was detected (Figure 4D), suggesting *cis*‐3MGC acid resists temperature‐induced isomerization to a greater extent than *trans*‐3MGC acid.

### 3.5 Effect of solvent composition or solution pH on 3MGC acid isomerization

**FIGURE 5:**
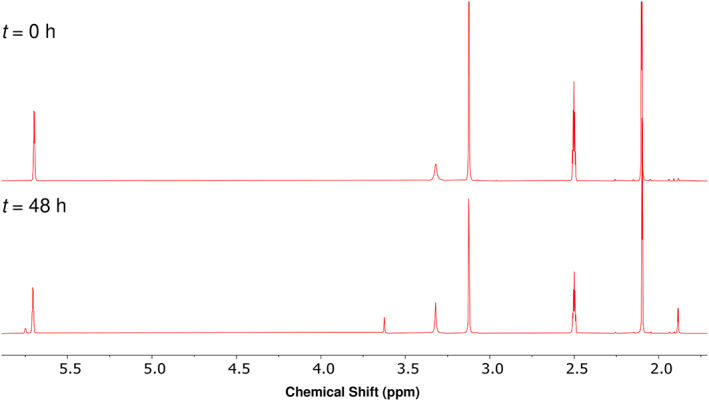
Effect of solvent on 3MGC acid isomerization. A sample of *trans*‐3MGC acid was dissolved in DMSO‐*d*_6_ and incubated for 0 or 48 hours at 25°C prior to analysis by ^1^H‐NMR spectroscopy. The ratio of *trans* and *cis* isomers present was determined by integration of the corresponding resonance peaks. Chemical shifts are in ppm units relative to the DMSO‐*d*_5_ solvent resonance at 2.5 ppm. The resonance at 3.32 ppm corresponds to residual H_2_O in the sample. New resonance peaks appearing in the sample incubated for 48 hours (5.75, 3.62, and 1.88 ppm) are specific for *cis*‐3MGC acid

^1^H‐NMR spectra of *trans‐* or *cis‐*3MGC acid in DMSO‐*d*_6_ at 25°C gave rise to three distinct proton resonances. Although the observed chemical shifts differ slightly from those observed in D_2_O, the relative positions of *cis‐* and *trans‐*3MGC acid chemical shifts were unchanged. When *trans*‐3MGC acid was dissolved in DMSO‐*d*_6_ and incubated at 25°C for 0 and 48 hours, respectively, the effect of incubation time on the diastereomeric ratio was assessed by ^1^H‐NMR spectroscopy (Figure 5). At 0 hour incubation no isomerization occurred. Aside from resonances for DMSO‐*d*_5_ (2.50 ppm) and residual H_2_O (3.32 ppm), resonances corresponding to the methyne proton (5.71 ppm), methylene protons (3.12 ppm) and methyl protons (2.10 ppm) were present. By contrast, after 48 hours, a total of six 3MGC acid‐ specific resonances were present. The new resonance peaks at 5.75, 3.62, and 1.88 ppm correspond directly to resonance peaks that appear in ^1^H‐NMR spectra of the *cis*‐3MGC acid standard in DMSO‐*d*_6_ (not shown). These data indicate that, compared to D_2_O, the activation energy for *trans‐*3MGC acid isomerization is lower in DMSO‐*d*_6_.

To assess the effect of solution pH on 3MGC acid isomerization, *trans‐* and *cis*‐3MGC acids were incubated in D_2_O at 70°C for 30 minutes under basic or acidic conditions. ^1^H‐NMR revealed both diastereomers experience a greater extent of isomerization when incubated under basic conditions than under acidic conditions (data not shown). When *trans*‐3MGC acid was incubated at 25°C under acidic or alkaline conditions, minimal isomerization was detected, as reported previously.^5^

## 4 DISCUSSION

The organic acid, 3MGC acid, is generated by thioester hydrolysis of 3MGC CoA. The biologically relevant diastereomer, *trans*‐3MGC CoA, is produced by one of two biosynthetic routes. In leucine metabolism 3MGC CoA is generated by carboxylation of 3‐methylcrotonyl CoA in a biotin‐dependent reaction catalyzed by 3‐methylcrotonyl CoA carboxylase. This reaction is stereospecific and exclusively generates *trans*‐3MGC CoA.^17^ When complete leucine metabolism is prevented by downstream pathway enzyme deficiencies, 3MGC CoA accumulates (primary 3MGC aciduria). An alternate route, termed the acetyl CoA diversion pathway,^2^, ^18^ occurs when mitochondrial energy metabolism is compromised by specific gene defects or disease processes (secondary 3MGC aciduria). Under these circumstances, as acetyl CoA accumulates in the matrix space it becomes a substrate for methylacetoacetyl‐CoA thiolase which functions in the reverse of its normal direction, condensing two equivalents of acetyl CoA to form acetoacetyl CoA. Acetoacetyl CoA reacts with another equivalent of acetyl CoA via HMG CoA synthase 2 to form (*S*)‐HMG CoA. Under these metabolic circumstances, HMG CoA serves as a substrate for AUH, which dehydrates (*S*)‐HMG CoA to *trans*‐3MGC CoA. In both primary and secondary 3MGC acidurias, as the concentration of *trans*‐3MGC CoA increases, it is generally considered to undergo thioester hydrolysis in a reaction catalyzed by a member of the acyl CoA thioesterase family,^19^ producing *trans*‐3MGC acid plus CoASH. Whereas *trans*‐3MGC acid is a dead‐end metabolite whose fate is excretion in urine, the liberated CoASH becomes available to maintain the mitochondrial pool of this key coenzyme.

A preferred method of analysis for 3MGC acid is GC‐MS.^7^ Interestingly, this method routinely yields a mixture of *cis‐* and *trans‐*3MGC acids. The ratio of *cis* and *trans* diastereomers observed by GC‐MS is variable, ranging from ~1:1 *cis:trans* to 2:1 *cis:trans*.^7^ Given that *trans*‐3MGC CoA is the sole diastereomer generated during metabolism, these data indicate isomerization occurs (a) naturally in vivo; (b) as an artifact of sample preparation procedures; or (c) a combination of both. Iles et al^4^ and Engelke et al^5^ used NMR spectroscopy to identify 3MGC acid in urine samples obtained from patients with primary 3MGC aciduria. In this case, evidence for the presence of *cis*‐ and *trans*‐3MGC acid was obtained, supporting the view that isomerization occurs in vivo. It is not known if *trans*‐3MGC CoA isomerizes to *cis*‐3MGC CoA in vivo, although indirect evidence, discussed below, suggests this reaction also occurs.

In the present investigation, *trans*‐ and *cis*‐3MGC acid standards were used in experiments designed to assess different aspects of the isomerization reaction. When either isomer was derivatized with TMS and subjected to GC‐MS, two peaks were present (see Figure 1). In keeping with previous studies,^20^ the earlier eluting peak was assigned as *cis*‐3MGC acid and the later eluting peak as *trans*‐3MGC acid. This assignment is consistent with the concept that intramolecular hydrogen bond formation in *cis*‐3MGC acid results in decreased interaction with the stationary phase.^21^

When samples of *trans*‐3MGC acid and *cis*‐3MGC acid were subjected to ^1^H‐NMR spectroscopy in D_2_O, a much different result was obtained. At 25°C, both diastereomers gave rise to spectra with little or no evidence of isomerization. Given that differences between the two methods of analysis include elevated temperature and TMS derivatization in the case of GC‐MS, an experiment was performed wherein *trans*‐3MGC acid and *cis*‐3MGC acid were subject to the GC‐MS preparation conditions without chemical derivatization. In this case, ^1^H‐NMR spectroscopy provided evidence of isomerization. Thus, it may be concluded that temperature and/or solvent are potential factors, but that TMS derivatization is not required for isomerization under these conditions. To further examine the effect of temperature on the isomerization reaction, *trans*‐3MGC acid and *cis*‐3MGC acid were subjected to various heating regimens followed by ^1^H‐NMR spectroscopy‐mediated determination of the diastereomeric ratio. It was observed that both *trans*‐ and *cis*‐3MGC acid undergo isomerization as a function of increasing temperature. However, under identical incubation conditions *trans*‐3MGC acid isomerized to a greater extent than *cis*‐3MGC acid. The results also reveal a correlation between incubation temperature and extent of isomerization. Moreover, the finding that incubation of *trans*‐3MGC acid at physiological temperature for days led to isomerization indicates this reaction has a relatively low energy of activation and occurs, albeit slowly, at physiological temperature. This result is consistent with the findings of Engelke et al^5^ who reported that urine obtained from subjects with primary 3MGC aciduria contains a mixture of *cis*‐ and *trans*‐3MGC acids.

**FIGURE 6:**
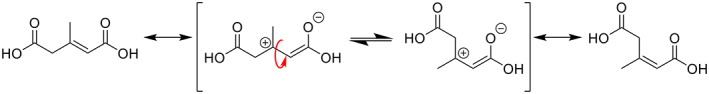
Proposed mechanism of 3MGC acid isomerization. Under appropriate conditions, the naturally occurring *trans* isomer of 3MGC acid (left) adopts a resonance intermediate wherein the double bond between C2 and C3 migrates to the C1‐C2 position, leaving a tertiary allylic carbocation at C3. The resonance structures (in brackets) allow free rotation around the C2‐C3 bond (see red arrow) such that subsequent re‐establishment of the C2‐C3 double bond results yields either *cis*‐3MGC acid (right arrow) or *trans*‐3MGC acid (left arrow)

A question that has not been addressed to date relates to the underlying mechanistic basis of 3MGC acid isomerization. In order for isomerization to occur, the C2‐C3 alkene must lose its double bond character to permit bond rotation. As shown in Figure 6, it is conceivable that the C2‐C3 double bond migrates to the C1‐C2 position due to electron withdrawing effects of the adjacent carboxylic acid combined with the inherent stability of the resulting tertiary allylic carbocation at C3. Once this resonance state forms, free rotation around the C2‐C3 bond is permitted. As the C1‐C2 alkene migrates back to the C2‐C3 position, either the *cis* or *trans* isomer will be produced.

A curious observation is that, not only does the ratio of *cis*:*trans* diastereomers in urine of subjects with 3MGC aciduria favor the *cis* diastereomer,^5^ the *cis* diastereomer displays greater resistance to isomerization than *trans*‐3MGC acid. The fact that *trans* configurations of double bonds generally confer stability to alkenes suggests some unique factor(s) may be responsible for the apparent higher stability of *cis*‐3MGC acid. Density functional calculations, that yielded energy‐minimized structures of *trans‐* and *cis*‐3MGC acid, indicated that, relative to each other, *trans*‐3MGC acid is 4 kJ/mol higher in energy. The lower energy of the *cis* diastereomer is consistent with the observations that (a) *cis*‐3MGC acid displays greater resistance to isomerization than *trans*‐3MGC acid and (b) biological samples analyzed by NMR spectroscopy manifest a higher percentage of *cis‐*3MGC acid.^5^

Thus, it appears that spontaneous isomerization of *trans*‐3MGC acid occurs slowly under in vivo conditions and this reaction contributes to the appearance of *cis*‐3MGC acid in urine of subjects with 3MGC aciduria.^5^ This mechanism, however, does not preclude the possibility of isomerization at the level of the acyl CoA. Indeed, an important structural difference between *cis‐* and *trans‐*3MGC CoA is their respective abilities to form a cyclic anhydride. Wagner et al^13^ reported that various acyl CoAs, including glutaryl CoA, 3‐methylglutaryl CoA and HMG CoA can undergo an internal rearrangement to form a cyclic anhydride with loss of CoASH. Whereas *trans*‐3MGC CoA is not capable of undergoing such an internal cyclization reaction, *cis*‐3MGC CoA is capable of cyclic anhydride formation via intramolecular nucleophilic attack of the thioester carbonyl by the carboxylate, with loss of CoASH. The observation by Wagner et al^22^ that protein 3MGCylation occurs via a reactive cyclic anhydride intermediate suggests that intramitochondrial isomerization of *trans*‐3MGC CoA takes place. Isomerization of *trans*‐3MGC CoA to *cis*‐3MGC CoA is a prerequisite for cyclic anhydride formation since neither the *trans* diastereomer of 3MGC CoA or *cis*‐3MGC acid will form the cyclic anhydride. Insofar as reaction of *cis‐*3MGC anhydride with H_2_O will generate *cis*‐3MGC acid, this reaction sequence could contribute to the relative proportion of *cis‐* and *trans‐*3MGC acid recovered in urine of subjects with 3MGC aciduria. Although Wagner et al^22^ reported that protein 3MGCylation occurs via a reactive anhydride intermediate, the relative reactivity of *cis*‐3MGC anhydride toward protein lysine side chains vs H_2_O is not known. In any case, protein lysine 3MGCylation can be reversed through the action of the NAD^+^ dependent deacylase, sirtuin 4,^23^ producing *cis*‐3MGC acid as product. Further studies will be required to elucidate the precise pathway(s) responsible for the appearance of a mixture of *cis* and *trans* diastereomers of 3MGC acid in urine of subjects with primary or secondary 3MGC aciduria.

## AUTHOR CONTRIBUTIONS

Dylan E. Jones conducted the experiments, analyzed the data, prepared the figures, and edited the manuscript. Juan David Ricker conducted molecular modeling experiments and edited the manuscript. Laina M. Geary contributed to the computational results data analysis and edited the manuscript. Dylan K. Kosma participated in GC‐MS experiments, organic acid derivatization, mass determinations, data interpretation, and manuscript editing. Robert O. Ryan conceived the project, designed experiments, interpreted data, and co‐wrote the manuscript.

## DISCLOSURE OF INTERESTS

The authors declare they have no competing interests.

## PATIENT CONSENT STATEMENT

Not applicable.

## DOCUMENTATION OF IACUC APPROVAL

Not applicable.

## Data Availability

The data that support the findings of this study are available from the corresponding author upon reasonable request.
